# Optimising the atrial fibrillation ablation pathway using vascular closure devices: effects on length of stay, procedure time and outcomes

**DOI:** 10.1007/s12471-026-02020-2

**Published:** 2026-03-03

**Authors:** Miriam A. Scheurwater, Bianca de Louw, Dennis van Veghel, Daniela N. Schulz, Alexandre J. Ouss, Rolf P. J. de Bruin, Lukas R. C. Dekker

**Affiliations:** 1 Catharina Heart Centre, Catharina Hospital Eindhoven, Eindhoven, The Netherlands; 2 Department of Biomedical Technology, Eindhoven University of Technology, Eindhoven, The Netherlands

**Keywords:** Atrial fibrillation, Catheter ablation, Vascular closure devices, Same-day discharge, Length of stay, Value-based healthcare

## Abstract

**Background:**

The rising prevalence of atrial fibrillation has led to increasing the numbers of pulmonary vein isolations. Optimising care pathway efficiency is essential for sustainable healthcare delivery. This study aimed to evaluate the impact of closure devices (CD) within the care pathway on hospital stay, patient satisfaction, staff workload, and costs, following catheter ablation for atrial fibrillation.

**Methods:**

This study compared the standard care pathway following catheter ablation using manual compression (MC, December 2023–February 2024) to a modified pathway incorporating suture-mediated CD (March-May 2024). Primary outcomes included process indicators (e.g., hospitalisation duration), clinical outcomes (e.g., bleeding complications), and patient experience. Secondary outcomes included staff experience and healthcare costs associated with procedural changes.

**Results:**

A total of 159 patients participated (MC: 81 patients, CD: 78 patients). Patients received an average of 2 percutaneous sutures. Treatment with CDs resulted in a 3.7-hour reduction in hospitalisation duration (*p* <. 001), 10-minute shorter procedure time in first-time ablations (*p* = .006), and 4‑hour shorter bed rest (*p* <. 001). Patients experienced less pain, measured using the Numeric Rating Scale (median 3 vs 1, *p* = .001), and used less pain medication (*p* = .006). Discharge comfort was higher in the CD group (*p* = .009), while complication rates remained similar. 24 nurses participated in the questionnaire. Most nurses reported improved time efficiency and workday organisation. Costs were similar across groups.

**Conclusion:**

CDs improve care pathways after atrial fibrillation ablation by reducing hospitalisation time, enhancing patient comfort, and improving workflow efficiency without increasing complications.

**Supplementary Information:**

The online version of this article (10.1007/s12471-026-02020-2) contains supplementary material, which is available to authorized users.

## What’s new

- This study confirms the value of using closure devices to optimize the care pathway for patients undergoing catheter ablation for atrial fibrillation.
- This approach significantly reduced hospitalisation duration, procedure time, and healthcare costs.
- Patients reported fewer pain complaints and greater discharge comfort.
- Optimizing the care pathway improved staff satisfaction through enhanced workflow efficiency and reduced workload due to shorter bed rest requirements.
- Continued research on procedural innovations aligned with VBHC-principles is essential to improve efficiency, cost-effectiveness, and patient-centred care in cardiac electrophysiology.

## Introduction

The prevalence of atrial fibrillation (AF) is projected to increase by 60% by 2050, increasing the burden on healthcare systems [1]. The economic impact is primarily driven by hospitalisations, heart failure, and thromboembolic events [2]. Current guidelines from the American College of Cardiology (ACC) and European Society of Cardiology (ESC) recommend catheter ablation with pulmonary vein isolation (PVI) to maintain sinus rhythm and reduce AF-related symptoms [3–5].

In many centres, post-ablation care involves manual femoral vein compression (MC), a pressure bandage, and ≥ 6 h of mandatory bed rest, with an additional one to two hours of rest to prevent puncture site complications [6, 7]. This approach often necessitates an overnight hospital stay, straining capacity, and increasing healthcare costs. Prolonged bed rest frequently causes physical discomfort (e.g., neck/back pain). Various closure devices (CDs) such as the collagen plug Vascade [7–9] and suture-mediated Perclose ProGlide device, [10–12] have been studied to achieve rapid haemostasis. While earlier studies suggest that CDs reduce hospitalisation duration [10–12], evidence is lacking regarding the impact on patient satisfaction, hospital staff workload, and logistics within cardiac catheterisation laboratories.

**Fig. 1:**
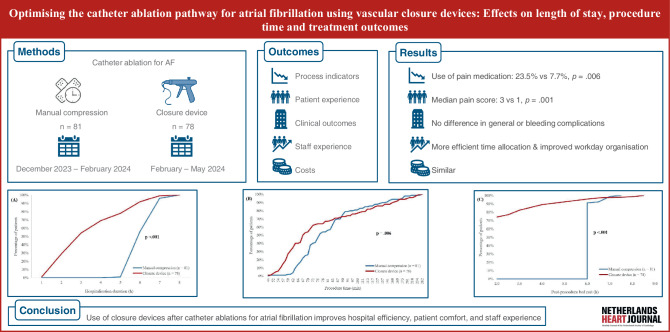
Infographic

This retrospective study examined process indicators, clinical outcomes, staff and patient experience in patients undergoing catheter ablation for AF with either MC or CD. The study focused on hospital stay, patient satisfaction, staff workload, and costs (Fig. 1).

## Methods

### Study setting

This study was part of a quality improvement project conducted at Catharina Heart Centre, Eindhoven, the Netherlands—a high-volume, non-academic hospital performing ~600 PVIs annually, including both primary and redo procedures. Consecutive patients treated between December 2023 and May 2024 were included. Data were collected prospectively during routine care, while the analysis was performed retrospectively after completion of both cohorts.

### Clinical protocols and population

Eligible patients (≥ 18 years, fluent in Dutch) undergoing catheter ablation for AF were included.

All patients underwent ultrasound-guided femoral vein catheter ablation with uninterrupted anticoagulation and activated clotting time > 300 s after transseptal puncture. For first-time catheter ablation, pulsed field ablation was conducted via a 16.8F sheath and a 6F sheath for a diagnostic catheter. Pulsed field ablation was conducted with the Farawave catheter (31 mm, Boston Scientific, Marlborough, MA, USA). Redo catheter ablation utilised 3D mapping systems (ENSITE or CARTO) via 10.7F and 12.1F sheaths. Pericardial effusion was routinely checked post-procedure using ultrasound.

From December 2023 to February 2024, MC was the standard for all consecutive patients, followed by six hours of pressure bandaging and bed rest, and two hours of observation before discharge.

From February to May 2024, CDs became the preferred groin management method. Patients were treated with two suture-mediated Perclose ProGlide devices (Abbott, Plymouth, MN, USA), followed by two hours of bed rest and one hour of observation. Discharge was permitted if no complications were observed. The goal was same-day discharge (SDD).

Eligible nurses were adults (≥ 18 years, cardiology ward staff) and were informed about the study by their department manager.

### Outcomes

Primary outcomes were process indicators (hospitalisation duration, procedure time, (prolonged) post-procedure bed rest, unplanned overnight stays, and pain medication use) and patient experience (pain ratings, pain-related complaints, and other complaints). Procedure time was defined as the duration from patient entry into the catheterisation laboratory to exit upon completion of the procedure. Unplanned overnight stays were defined as unscheduled hospital admissions following the procedure and were calculated as a proportion of patients who were not planned for overnight observation. Planned overnight stays, which occurred more frequently in the MC group, were not considered part of this outcome. Process indicators were extracted from electronic medical records.

Patient experience was assessed through a 14-question survey on pain, information delivery, nursing care, discharge process, and an overall hospital stay rating (Supplementary materials 1). The questionnaire was developed by the study team and reviewed by experts. Pain was measured using the Numeric Rating Scale (NRS) [13].

Secondary outcomes focused on clinical outcomes (general and bleeding complications), staff experience, and costs. General complications were defined as hypotension, respiratory desaturation, and neurological complaints (e.g., coordination disorders). Bleeding complications were classified using the Bleeding Academic Research Consortium system, [14]^,^ though types 3a, 3b, and 4 could not be subtyped due to missing haemoglobin measurements. Clinical outcomes were extracted from electronic medical records.

Staff experience was assessed through a 21-question nurse-reported survey on perceived workload, task changes, and confidence in SDD (Supplementary materials 2). The survey was developed by the study team and reviewed by experts.

Hospitalisation costs were obtained from the Dutch Healthcare Authority (Nederlandse Zorgautoriteit) Open Data portal, which provides national average prices of all healthcare services, using 2024 price levels [15]. These encompass the full care episode from admission to discharge, including procedural costs. The cost of CDs was calculated using the average Dutch price (€ 150 per device). Personnel costs for catheterisation laboratory and nursing staff was derived from the Dutch national collective labour agreement, respectively € 4842/month (group 55, grade 12) and € 4324/month (group 50 grade 12) [16]. Cardiologists’ income was set at € 150/hour. The analysis aimed to evaluate whether the use of CDs, despite their additional material cost, resulted in a change in total hospitalisation costs per patient compared to the MC group.

### Baseline patient characteristics

Baseline characteristics were defined using the definitions of the Netherlands Heart Registration, [17]^,^ which align with the ESC/ACC and American Heart Association guidelines [18]. Extracted variables from electronic medical records included: age, gender, body mass index (BMI), paroxysmal, persistent, or unknown AF, left atrial volume index, previous ablation, CHA_2_DS_2_-VASc score, medical history (i.e., cerebrovascular accident, diabetes, hypertension, myocardial infarction, obstructive sleep apnoea, peripheral artery disease, and renal insufficiency), and type of oral coagulant.

### Access-site and operator characteristics

The following procedural characteristics were extracted from electronic medical records: operator, CD procedural success, entry and exit times in the catheterisation laboratory, reaccess of the femoral vein, and any related access difficulties.

### Statistical methods

Data analysis was performed using SPSS version 29 (IBM, Chicago, IL, USA). Normally distributed data were presented as mean ± SD, non-normally distributed data as median (IQR).

The MC group was compared with the CD group by using Fisher’s exact/Chi-square tests for categorical variables, unpaired T‑test for normally distributed numeric data, and Mann-Whitney U test for non-normally distributed data. Data distribution was assessed with histograms and Q‑Q plots.

To account for potential bias related to hospitalisation duration, the interval between hospital admission and procedure start was compared between groups. Additionally, a subgroup analysis was performed to compare procedure time between groups separately for first-time and redo ablations.

Regression analyses were performed to assess associations between baseline characteristics and primary and secondary outcomes. Additionally, the association between oral coagulant type and operator was examined. Linearity was assessed using scatter plots. The Hosmer-Lemeshow test was used to assess the logistic regression model fit. A *p*-value *p* < 0.05 was considered statistically significant.

Eight items of the nursing questionnaire were included in the Cronbach’s alpha; other measured distinct constructs or were unsuitable due to a categorical or open-ended format. Therefore, the mean inter-item correlation was also evaluated.

Mean costs in the MC- and CD group were compared, and average costs savings per patient were noted.

### Ethical considerations

The study followed the Declaration of Helsinki, was approved by the local ethics committee, and participation was voluntary.

## Results

### Participants

A total of 159 patients were included (81 MC, 78 CD, Tab. 1). Mean age was 65.0 ± 8.5 years, without a significant difference between groups (*p* = 0.166). Males comprised 66.7% of the total population (65.4% in the MC group vs. 67.9% in the CD group, *p* = 0.866). Persistent AF was present in 25.9% of the MC group compared to 50% in the CD group, while paroxysmal AF was observed in 72.8% of the MC group versus 46.2% in the CD group (*p* = 0.002). No other baseline characteristics showed significant differences.

**Table 1:** Baseline characteristics

	Total population (*n* = 159)	Manual compression (*n* = 81)	Closure device (*n* = 78)	*p*-value
Age, years, mean ± SD	65.0 ± 8.5	64.2 ± 9.3	64.8 ± 7.6	0.166
Gender, male, *n* (%)	106 (66.7%)	53 (65.4%)	53 (67.9%)	0.866
BMI, kg/m^2^, mean ± SD	27.2 ± 3.8	27.4 ± 3.6	27.0 ± 4.1	0.711
*Type of AF, n (%)*				**0.002**
Persistent	60 (37.7%)	21 (25.9%)	39 (50.0%)	
Paroxysmal	95 (59.7%)	59 (72.8%)	36 (46.2%)	
Unknown	4 (2.5%)	1 (1.2%)	3 (3.8%)	
*LVEF, n (%)*				0.227
Normal	131 (83.4%)	70 (87.4%)	61 (79.2%)	
Mildly reduced	15 (9.6%)	5 (6.3%)	10 (13.0%)	
Moderate reduced	9 (5.7%)	5 (6.3%)	4 (5.2%)	
Severe reduced	2 (1.3%)	0	2 (2.6%)	
LAVI, mean ± SD	38.5 ± 11.8	38.7 ± 12.7	38.3 ± 11.1	0.754
Previous ablation, *n* (%)	42 (26.4%)	22 (27.2%)	20 (25.6%)	0.828
CHA_2_DS_2_-VASc score ≥ 2, *n* (%)	85 (53.5%)	45 (55.6%)	40 (51.3%)	0.635
*Medical history, n (%)*				
CVA	9 (5.7%)	6 (7.4%)	3 (3.8%)	0.496
Diabetes	8 (5.0%)	4 (4.9%)	4 (5.1%)	1.000
Hypertension	45 (28.3%)	21 (25.9%)	24 (30.8%)	0.498
Myocardial Infarction	5 (3.1%)	2 (2.5%)	3 (3.8%)	0.678
OSA	7 (4.4%)	5 (6.2%)	2 (2.6%)	0.433
PAD	0	0	0	−
Renal insufficiency	1 (0.6%)	1 (1.2%)	0	1.000
*Type of anticoagulant, n (%)*				0.462
Direct oral anticoagulants	154 (96.9%)	79 (97.5%)	75 (96.2%)	
Vitamin K antagonists	5 (3.1%)	2 (2.5%)	3 (3.8%)	

*AF* atrial fibrillation, *BMI* body mass index, *CVA* cerebrovascular accident, *LAVI* left atrial volume index, *LVEF* left ventricular ejection fraction, *OSA* obstructive sleep apnoea, *PAD* peripheral artery disease, *SD* standard deviation

### Process indicators

**Fig. 2:**
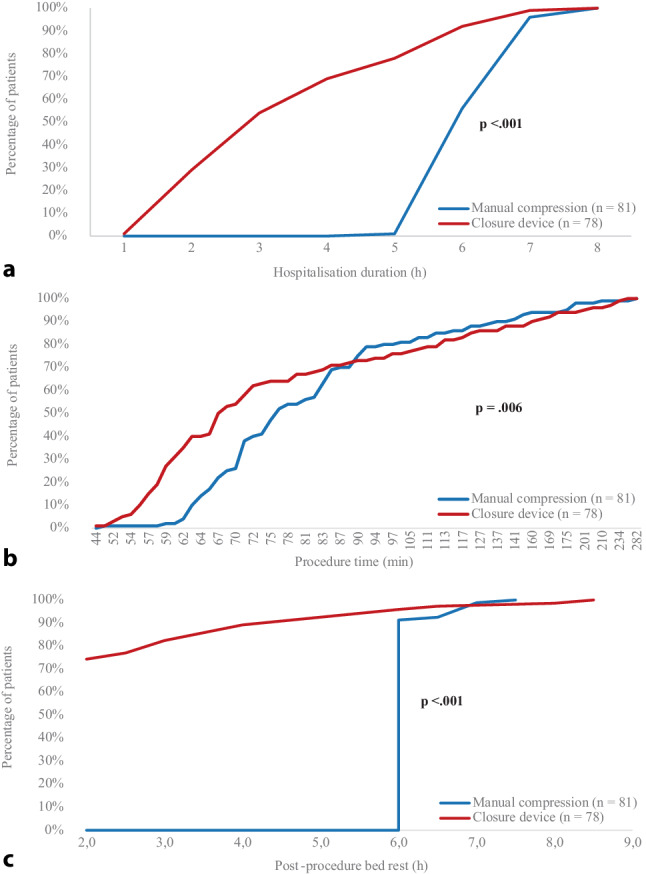
Cumulative percentage of patients with (**a**) hospitalisation duration in hours, (**b**) procedure time in minutes, and (**c**) hours of bed rest

Hospitalisation duration was shorter in the CD group (7.9 vs. 11.6 h, *p* <. 001, Tab. 2). There was no significant difference in the interval between hospital admission and procedure start between groups, indicating that procedure timing did not influence hospitalisation duration. Procedure time was shortened by 10 min (*p* = 0.006) and post-procedure bed rest decreased from 6 to 2 h (*p* <. 001, Fig. 2). When analysed separately, procedure time remained significantly shorter in the CD group for first-time ablations (*p* <. 001), but not for re-do ablations (*p* = 0.442). Prolonged bed rest was more common in the CD group (19 vs. 5 patients, *p* = 0.007), but unplanned overnight stays did not increase. Similarly, patients in the CD group received less pain medication (*p* = 0.006).

**Table 2:** Process indicators, patient experience, and clinical outcomes

	Manual compression (*n* = 81)	Closure device (*n* = 78)	*p*-value
*Process indicators*			
Hospitalisation duration in hours, median (IQR)	11.6 (11.1–12.9)	7.9 (6.9–9.6)	**<** **0.001**
Procedure time in minutes, median (IQR)	77.0 (69–91)	67.5 (59–99)	** 0.006**
Post-procedure bed rest in hours, median (IQR)	6.0 (0)^*^	2.0 (2.0–2.5)	**<** **0.001**
Prolonged post-procedure bed rest, *n* (%)	5 (8.1%)	19 (25.7%)	** 0.007**
Unplanned overnight stay, *n* (%)^**^	5 (6.6%)	1 (1.3%)	0.210
Use of pain medication, *n* (%)	19 (23.5%)	6 (7.7%)	** 0.006**
*Patient experience*			
Pain score^†^, median (IQR)	3 (0–6)	1 (0–4)	** 0.001**
Pain complaints, total, *n* (%)	53 (65.4%)	37 (47.4%)	** 0.026**
Abdominal pain	5 (6.2%)	5 (6.4%)	1.000
Chest pain	16 (19.8%)	10 (12.8%)	0.237
Groin pain	13 (16.1%)	15 (19.2%)	0.599
Headache	14 (17.3%)	6 (7.7%)	0.068
Neck or back pain	31 (38.3%)	7 (9.0%)	**<** **0.001**
Pain in arms and/or legs	4 (4.9%)	2 (2.6%)	0.682
Other	8 (9.9%)	4 (5.1%)	0.370
Other complaints, total, *n* (%)	21 (26.3%)	26 (34.2%)	0.279
Anxiety/stress	6 (7.5%)	6 (7.9%)	0.926
Dizziness	9 (11.3%)	10 (13.2%)	0.716
Dyspnoea	1 (1.3%)	4 (5.3%)	0.201
Nausea/vomiting	7 (8.8%)	4 (5.3%)	0.535
Other	4 (5.0%)	8 (10.5%)	0.238
Comfortable with discharge, *n* (%)	67 (82.7%)	75 (96.2%)	** 0.009**
Rating of patient care unit, median (IQR)	9.0 (8.0–10.0)	9.0 (8.0–10.0)	0.458
*Clinical outcomes*			
General complications, *n* (%)^‡^	5 (6.2%)	12 (15.4%)	0.060
Bleeding complications, total, *n* (%)^§^	24 (29.6%)	16 (20.5%)	0.185
BARC 1	13 (16.0%)	6 (7.7%)	0.104
BARC 2	11 (13.6%)	11 (14.1%)	0.924
BARC 3c	0	0	–
BARC 5	0	0	–

*BARC* Bleeding Academic Research Consortium, *IQR* interquartile range

Statistically significant *p*-values are shown in bold. *The IQR of 0 h in the manual compression group reflects that the majority of patients followed the protocol-defined 6 h bed rest, and only 5 patients (8.1%) required prolonged bed rest (> 6 h). **Percentages for unplanned overnight stays were calculated among patients without a planned overnight observation. † Pain scores were measured using the Numeric Rating Scale (range 0–10). ‡ General complications are defined as hypotension, respiratory desaturation, and neurological complaints (e.g. coordination disorders). § Bleeding complications are defined as any sort of bleeding, including haematoma.

### Patient experience

CD patients reported lower pain scores (median 3 vs 1, *p* = 0.001), especially less neck and back pain (*p* < 0.001). No differences were observed in other complaints (e.g., nausea). CD patients reported greater discharge comfort (*p* = 0.009). There was no difference in patients’ overall experience on the cardiology ward.

### Clinical outcomes

No differences were observed in general or bleeding complications. Age, gender, and BMI were not associated with any of the outcomes. Previous ablation was only associated with longer procedure time (B = 69.5 min, *p* <. 001). There was no association between oral coagulant type or operator and any of the outcomes (Supplementary materials 3).

### Staff experience

A total of 24 nurses participated in the questionnaire (30.8% response rate). The internal consistency of the questionnaire was moderate (α = 0.664; mean inter-item-correlation = .205).

Demographics of responding nurses are displayed in supplementary materials 4. Most were aged between 21–29 years (54.2%) and female (75.0%) with over five years of nursing experience.

Nursing staff routines changed due to reduced bed rest, enabling earlier patient mobilisation and more synchronised admission and discharge times. The advantages and disadvantages are displayed in Tab. 3. Nine nurses noted occasional workload peaks, and six found this disruptive. Overall, 45.8% reported no disadvantages.

**Table 3:** Reported impact of closure devices as reported by nurses

Perceived Impact	*n* (%)
*Advantages*	
Faster patient mobility	23 (95.8%)
More efficient nursing time allocation	17 (70.8%)
Improved workday organisation	15 (62.5%)
*Disadvantages*	
Increased stress	2 (8.3%)
Higher workload perception	2 (8.3%)
Disruptive occasional peaks in workload	6 (25.0%)
Greater care burden	1 (4.2%)

Optimising daily organisation accelerated patient flow, reducing the need for evening shift nursing staff and enabling the reallocation of one nurse.

### Cost estimation

Despite the investments in CDs, average overall costs per patient remained similar across groups (€ 11,763.89 in the MC group vs € 11,679.19 in the CD group) (Supplementary materials 5). This cost neutrality indicates that the higher material costs were offset by shorter procedure times, higher SDD rates (86.4% to 98.7%), and corresponding reductions in catheterisation laboratory personnel (€ 48.83) and nursing (€ 42.56) costs per patient. Savings related to reduced staff time were based on the shorter procedure time and reduced nursing hours, multiplied by staff costs per hour. Although not included in this analysis, the reduction in procedure time may allow for higher procedural capacity and potential revenue gains.

## Discussion

In this retrospective study, the effects of introducing CDs after catheter ablation for patients with AF were evaluated with respect to process indicators, clinical outcomes, patient and staff experience, and costs. The aim of this quality improvement project was to minimise the length of hospital stay without compromising patient satisfaction, while reducing hospital staff workload and costs. Our findings showed that the use of CDs resulted in shorter hospitalisation duration, shorter procedure time for first-time ablations, fewer pain complaints, reduced pain medication needs, and similar costs per patient. Additionally, nurses reported improved nursing time allocation and workday organisation.

These findings align with Value-Based Healthcare (VBHC) principles, which prioritise optimising patient outcomes, cost efficiency, and resource utilisation [19–21]. Implementing CDs in standard practice may improve workflow efficiency and healthcare delivery, while ensuring financial sustainability and not increasing complications.

Our results corroborate previous studies demonstrating reduced pain complaints with shorter bed rest and decreased pain medication use [7]. Earlier research indicated that shorter bed rest after treatment with a CD was not associated with higher readmission rates, supporting the safety of early mobilisation [6]. While this study was not powered to address complications, no differences were observed in general or bleeding complications, aligning with existing literature [9, 11, 12].

Unlike a previous study by Mohanty et al. [7], where no CD patients required more than two hours of bed rest, this study included patients on extended bed rest, mainly for small haematomas. Despite limited clinical relevance, bed rest was extended as a precaution, possibly reflecting cautious implementation. The decision was made by nursing staff, which may have differed if discussed with the operator.

Patient satisfaction was comparable between both groups, unlike the study by Natale et al., [9]^,^ which reported higher satisfaction in the CD group. This discrepancy may be attributed to our already high patient satisfaction scores, making it difficult to detect differences. Differences in patient-reported outcomes may have been influenced by the questionnaire used, as Natale et al. included a comparative question for patients with prior ablation experience.

In our centre, MC followed by pressure bandaging and bed rest was standard care. The figure-of‑8 suture, regularly used in other centres to achieve haemostasis, was not part of our protocol due to low complication rates with MC and the concurrent transition to CDs.

A key strength of this study is its alignment with VBHC principles. CDs reduced procedure time, hospitalisation duration, and pain complaints without increasing complications, indicating increased clinical en economical value. Additionally, while not the primary focus of VBHC, improved workflow efficiency and staff satisfaction may support broader sustainability goals by enhancing staff retention and care delivery capacity. From a patient-centred perspective, prioritising comfort, safety, and recovery experience remains essential in high-value procedural care.

Limitations include the observational design and retrospective data analysis; however, baseline characteristics were comparable between groups, minimising confounding by indication. Patient-reported outcomes may have been influenced by response bias, as nurses informed patients that CDs represent a care innovation, potentially affecting their perception of discharge readiness. Finally, the cost analysis used national hospitalisation costs and excluded potential revenue from increased procedural capacity, suggesting actual savings may be greater.

Future research should refine economic evaluations to better quantify the cost-effectiveness and reimbursement implications of CDs across different healthcare settings, and utilise benchmark data to compare outcomes across institutions and develop quality improvement initiatives. Significant potential remains for further optimisation of CDs to improve safety and usability, leading to better patient outcomes. Continued research on procedural innovations aligned with VBHC principles is essential to improve efficiency, cost-effectiveness, and patient-centred care in cardiac electrophysiology.

## Conclusion

This study demonstrates that treatment with CDs after AF catheter ablations improves hospital efficiency, patient comfort, and staff experience. Future research should compare outcomes across institutions using benchmark data to further develop quality improvement initiatives, focus on cost-effectiveness across diverse healthcare settings, and explore advancements in CD technology to enhance safety, usability, and overall patient outcomes.

## Supplementary Information

ESM1: Supplementary material 1
