# Supplementary material for: Optimising the atrial fibrillation ablation pathway using vascular closure devices: effects on length of stay, procedure time and outcomes
Source: Neth Heart J. 2026 Mar 3;34(4):133–42. doi: 10.1007/s12471-026-02020-2 (PMC13009434; doi:10.1007/s12471-026-02020-2)
Supplement: Supplementary file 1 — ESM1: Supplementary material 1 [file 12471_2026_2020_MOESM1_ESM.docx]

**Supplementary materials**

**S1. Patient questionnaire**

Notably, the original questionnaire was provided in Dutch.

Dear Sir/Madam,

You were admitted to the cardiac care department today and underwent a procedure. We are interested in your experience. Could you please answer the following questions?

Patient number ……………….. (to be completed by the nurse)

Ablation date ………………….

1. Did the nurse provide you with sufficient information about your admission and procedure upon arrival?

☐ Yes
☐ No
Additional comments:……………..

2. How many hours of bed rest did you have after your procedure?

……. hours

3. Were you able to go to the toilet independently after the procedure?

☐ Yes, I was allowed to walk to the toilet
☐ Yes, but I was taken to the toilet in a wheelchair
☐ No, I had to use a bedside commode
☐ No, I had to use a bedpan
☐ No, I was catheterised once
☐ Not applicable

4. Did you experience any pain during your stay at the cardiac care department? (multiple answers possible)

☐ Yes, neck or back pain
☐ Yes, abdominal pain
☐ Yes, pain in arms or legs
☐ Yes, headache
☐ Yes, groin pain
☐ Yes, chest pain
☐ Yes, other: …………………………………
☐ No

5. On a scale of 0-10, how severe was your pain at its worst?

0, no pain

1  6

2  7

3  8

4  9

5  10, very severe pain

6. Did the nurse pay attention to your pain complaints?

☐ Yes
☐ No
☐ I did not report my pain complaints to the nurse
☐ I had no pain complaints

7. Did you receive any medication for the pain?

☐ Yes, specify: ……………………
☐ No
☐ Not applicable

8. Did you experience any other complaints during your stay at the cardiac care department? (multiple answers possible)

☐ Yes, nausea and/or vomiting
☐ Yes, anxiety or tension
☐ Yes, shortness of breath or difficulty breathing
☐ Yes, dizziness
☐ Yes, other: ………………………………
☐ No

9. This procedure is intended as day treatment. Were you informed about the reason for an extended hospital stay?

☐ Yes
☐ No
☐ Not applicable, I was discharged the same day

10. I felt confident enough to go home

☐ Strongly agree
☐ Agree
☐ Neutral
☐ Disagree
☐ Strongly disagree
☐ Not applicable

11. Upon discharge, did you receive information about your medication use after hospitalisation?

☐ Yes
☐ No
☐ Not applicable, I did not start (new) medication

12. Upon discharge, did you receive information on what to do if problems arise after leaving the hospital?

☐ Yes
☐ No

13. How would you rate your stay at the cardiac care department?

☐ 1, very poor
☐ 2
☐ 3
☐ 4
☐ 5
☐ 6
☐ 7
☐ 8
☐ 9
☐ 10, excellent

14. Do you have any additional comments?

………………………..

Thank you very much for completing this questionnaire.

**S2. Nursing staff questionnaire**

Notably, the original questionnaire was provided in Dutch.

Dear Colleague,

Since March 2024, the new closure device protocol has been implemented, allowing patients to have a shorter bed rest period after ablation due to the use of a closure device. Previously, the 6:1:1 method was used, which included six hours of bed rest.

We are interested in your impressions of this new approach and kindly ask you to complete the questionnaire below.

1. What is your gender?

☐ Male
☐ Female
☐ Prefer not to say

2. What is your age?

………………………….

3. How many years have you been working as a nurse?

………………………….

4. How many years have you been working in the cardiology department at Catharina Hospital?

………………………….

5. Did you receive prior instructions on caring for a patient treated with a closure device? If so, did you find the instructions sufficient?

☐ Yes
☐ No, please specify: …………………..

6. Have you observed any specific problems or challenges in patients treated with a closure device? If so, what were they?

☐ No problems observed
☐ Pain at the groin and/or puncture site
☐ Leakage from the dressing
☐ Bleeding
☐ Soft swelling at the puncture site
☐ Hard swelling at the puncture site
☐ Other:……………………..

7. I feel competent in caring for a patient treated with a closure device.

☐ Strongly agree
☐ Agree
☐ Neutral
☐ Disagree
☐ Strongly disagree
Additional comments:………………………..

8. Compared to the previous method, I find patient comfort with the new approach to be:

☐ Much better
☐ Better
☐ Neutral
☐ Worse
☐ Much worse
Please explain your answer:………………….

9. Compared to the previous method, I find the patient care workload to have:

☐ Increased significantly
☐ Increased
☐ Remained the same
☐ Decreased
☐ Decreased significantly
Additional comments:………………………

10. In my opinion, the use of pain medication in patients treated with a closure device has:

☐ Increased significantly
☐ Increased
☐ Remained the same
☐ Decreased
☐ Decreased significantly
Additional comments:……………………………….

11. I feel confident in discharging patients after a shorter observation period when the groin is closed with a closure device.

☐ Strongly agree
☐ Agree
☐ Neutral
☐ Disagree
☐ Strongly disagree
Additional comments:…………………

12. What benefits do you see in using a closure device? (Multiple answers possible)

☐ Faster mobilisation
☐ Less pain complaints (e.g., back pain)
☐ Shorter hospital stay
☐ Improved patient satisfaction
☐ More efficient use of nursing time
☐ Increased number of procedures per day
☐ More efficient workday organisation
☐ Other, namely:……………………
☐ I see no benefits

13. What drawbacks do you see in using a closure device? (Multiple answers possible)

☐ Increased workload
☐ Increased patient care demands
☐ Higher stress levels among nurses due to shorter observation period
☐ Increased peak moments due to simultaneous tasks
☐ Other, namely:………………………..
☐ I see no drawbacks

14. If a closure device allows for a more efficient workday, how would you prefer to use this additional time?

☐ Spend more time on direct patient care, such as providing emotional support or performing more thorough assessments
☐ Participate in professional development activities, such as workshops or training to improve nursing skills and knowledge
☐ Update existing protocols
☐ A closure device does not create a more efficient workday because: …………………..
☐ Other, namely:……………………….

15. I experience simultaneous tasks occurring on the ward, known as peak moments, as:

☐ Very unpleasant
☐ Unpleasant
☐ Neutral
☐ Pleasant
☐ Very pleasant

16. Compared to the previous method, peak moments have:

☐ Increased significantly
☐ Increased
☐ Remained the same
☐ Decreased
☐ Decreased significantly

17. Compared to the previous method, I experience the patient flow in the cardiac care department as:

☐ Very positive
☐ Positive
☐ Neutral
☐ Negative
☐ Very negative
Additional comments:…………………….

Thank you for your time and participation!

**S3. Associations of selected clinical outcomes**

**Supplementary table 1.** Associations between type of anticoagulant and operator and the occurrence of general or bleeding complications.

|  | **General complications** | ***p*-value** | **Bleeding complications** | ***p*-value** |
| --- | --- | --- | --- | --- |
| Type of anticoagulant, n (%)  Direct oral anticoagulants  Vitamin K antagonists | 16 (10.4%)  1 (20%) | .672 | 39 (25.3%)  1 (20.0%) | .514 |
| Operator  1  2  3  4  5  6  7 | 2 (12.5%)  1 (4.0%)  2 (6.1%)  0  6 (15.8%)  4 (20%)  2 (14.3%) | .355 | 5 (31.5%)  5 (20.0%)  10 (30.3%)  3 (23.1%)  6 (15.8%)  8 (40.0%)  3 (21.4%) | .501 |

**S4 Demographics of responding nursing staff**

**Supplementary table 2.** Demographic characteristics of responding nursing staff.

| **Characteristic** | **n (%)** |
| --- | --- |
| **Age (years)** |  |
| 21 – 29 | 13 (54.2%) |
| 30 – 39 | 5 (20.8%) |
| 40 – 49 | 2 (8.3%) |
| 50 – 59 | 4 (16.7%) |
| **Gender** |  |
| Female | 18 (75.0%) |
| **Nursing Experience (years)** |  |
| 0 – 1 | 4 (16.7%) |
| 1 – 3 | 7 (29.2%) |
| 3 – 5 | 4 (16.7%) |
| >5 | 9 (37.5%) |

**S5. Cost analysis**

**Supplementary table 3**. Cost analysis for the manual compression and closure device group.

|  | | **Manual compression** | | **Closure device** | |
| --- | --- | --- | --- | --- | --- |
| **Cost parameter** | **€ per patient** | **N. of patients** | **Total €** | **N. of patients** | **Total €** |
| Hospitalisation costs with nightstay 2024* | €13,825 | 11 | €152,075 | 1 | €13,825 |
| Hospitalisation costs with same day discharge 2024† | €11,440 | 70 | €800,800 | 77 | €880,880 |
| **Additional costs and benefits** | | | | | |
| Closure devices | €300 | 0 | - | 78 | €23,400 |
| Reduction hours catheterization laboratory personnel | €48.83 | 0 | - | 78 | -€3808.74 |
| Reduction nursing hours cardiology ward | €42.56 | 0 | - | 78 | -€3319.68 |
| **Total** | €952,875.00 | | | €910,976.58 | |
| **Total per patient** | **€11,763.89** | | | **€11,679.19** | |

* Calculated using the national average sale price for a Class 4 procedure to treat cardiac arrhythmias in the context of a heart or lung condition during hospitalisation in 2024 (number 979001233), extracted from the Open Data portal of the Dutch Healthcare Authority.^[15]^
† Calculated using the national average sale price for a Class 4 procedure to treat cardiac arrhythmias in the context of a heart or lung condition in 2024 (number 979001234), extracted from the Open Data portal of the Dutch Healthcare Authority.^[15]^
